# Breast cancer treatment and recovery: pets’ roles as emotional buffers and stressors

**DOI:** 10.1186/s12905-023-02662-z

**Published:** 2023-10-17

**Authors:** Lori R. Kogan, Jennifer Currin-McCulloch, Linda S. Cook

**Affiliations:** 1 Clinical Sciences Department, Colorado State University, 1680 Campus Delivery, Fort Collins, CO 80523 USA; 2 School of Social Work, Colorado State University, 1586 Campus Delivery, Fort Collins, CO 80523 USA; 3 Colorado School of Public Health, CU Anschutz Medical Campus, Building 500, 13001 East 17Th Place, B119, Aurora, CO 80045-2601 USA

**Keywords:** Breast cancer survivorship, Pets, Guilt, Support, Stressors

## Abstract

**Background:**

Research suggests pets foster affection, connection, and physical activity, yet has failed to address the challenges people diagnosed with cancer face in caring for their pets. The objective of this study was to better understand how pets serve as emotional buffers and/or stressors for people diagnosed with breast cancer, and how their ability to meet their pet’s needs affects their well-being.

**Methods:**

A cross-section study of people diagnosed with breast cancer in the United States was conducted. Adults diagnosed with stages 0 (in situ) -IV breast cancer and currently the primary guardian of at least one dog or cat and owned the animal(s) for at least 6 months, were recruited for the study. A total of 211 responses, obtained between July – November 2022 were analyzed. The survey included questions about participants’ demographics; attachment to their pets; physical, emotional, and functional well-being; social support received from their pet; and ‘pet parenting’ concerns. Descriptive statistics were calculated to describe participants’ demographics. Multiple regression analyses were conducted to determine predictors of pet attachment, well-being, support from pet, and ‘pet parenting’ concerns.

**Results:**

People diagnosed with breast cancer derive substantial support from their pets (80% feel their pet makes them feel loved, needed, and offers a positive presence in the home), yet only 50% of participants feel this relationship is supported by their medical team. Controlling for owner demographics, heightened levels of pet-related guilt and concerns, along with lower perceived support from their pet, are all significant predictors of a lower quality of life.

**Conclusions:**

Findings highlight the benefits pets offer people diagnosed with breast cancer, yet also the distress they feel in trying to meet their pet’s needs. Assessment conversations about pet ownership, including pet-related support systems, are needed to validate people’s concerns and support the identification and development of pet support teams. Medical team facilitated discussions about pet care needs is suggested to demonstrate support for the pet-parent bond and help normalize feelings of guilt related to challenges in meeting their pet’s needs. These discussions could be aided through the development of research-driven intervention strategies and online, freely accessible targeted tools.

**Supplementary Information:**

The online version contains supplementary material available at 10.1186/s12905-023-02662-z.

## Background

### Breast cancer

Breast cancer is the most common cancer in women, with an expected 290,560 new cases in the United States (U.S.) in 2022 [1]. Of the estimated 18.1 million cancer survivors in the U.S., an estimated 4.1 million (22%) are female breast cancer survivors, representing the largest group of survivors by cancer type [2]. This large and growing group of breast cancer survivors highlights the importance of research that continues to address unmet needs in health-related quality of life and wellbeing [3].

Regardless of the stage of breast cancer diagnosis, the physical, psychosocial and spiritual distress associated with this potentially life-limiting illness create rippling effects throughout a person’s life, as well as that of their support system. The onslaught of medical appointments, inpatient surgeries, outpatient procedures, and treatments place women at risk for a host of physical [4–6] and psychosocial side effects [7, 8] creating obstacles for people to sustain their previous quality of life. For example, postoperative orders following breast surgery require that they lift no more than five pounds and not perform any repetitive arm movements on the side where they had breast surgery [9]. These factors subsequently restrict the ability to complete personal care, let alone support others living in their home. Furthermore, nearly all household duties must be done by other family members or friends [10]. As a result, people living alone, or with minimal instrumental support, face additional burdens in securing support to perform postoperative personal care and household chores [11].

### Emotional labor

In their roles as intimate partners and/or parents, people diagnosed with breast cancer have additional tasks to navigate at the time of surgery and during chemotherapy, radiation therapy, and hormonal therapy. Gender socialized roles presume that female-identifying persons will fulfill the majority of the emotional labor for their families [12] and their inability to perform emotional surveillance and care places women at risk for emotional distress [10, 11]. Relinquishing duties to others or asking for help can exacerbate these distressing feelings, as this goes against socialization that women must be able to manage everything [11].

Breast cancer survivors and parents experiencing other types of cancer report family-specific psychosocial supportive care needs for themselves as a parent (e.g., support regarding parenting concerns) as well as needs regarding practical aspects (e.g., childcare, household help) [13]. Young mothers with cancer frequently question their ability to be “a good parent” [14] and may experience anxiety, depression, and guilt [15, 16]. People living with metastatic breast cancer may experience emotional distress surrounding their potential untimely death and the negative impacts this may have on their child’s well-being [16–18]. They may also worry about the psychosocial well-being and instrumental care for others in their home including their partners, children, and companion animals.

### Pet attachment

Having a pet in the U.S. is the norm; approximately 70% of U.S. homes include at least one pet [19]. Pets have been shown to provide a wide array of benefits including increased wellbeing [20–26], stress reduction [27–29], a buffer against depression [30–34] and anxiety [35–37], and a buffer against stressors associated with the COVID-19 pandemic [38–43]. It should be noted, however, that some studies have failed to find positive physical or psychological effects of pets [44–46]. This may be due to the nuances between pet ownership and pet attachment. While a minority of people merely own a pet, most pet owners (85% of dog owners and 76% of cat owners) consider their pets to be family members [47]. For this reason, it can be helpful to view the pet/owner relationship through an attachment theory lens.

Originally, the concept of attachment was used to conceptualize child–parent relationships [48, 49], but it has since expanded to include human–pet relationships. Attachment can be defined as an enduring relationship with a particular other that results in one individual seeking and maintaining proximity to the object of their attachment, displaying confident behavior in the presence of the attachment figure, and showing distress upon involuntary separation [48]. Attachment theory can be applied to relationships that fulfill four criteria: (a) proximity maintenance – preferring to be near an attachment figure, especially in times of stress or need; (b) using the attachment figure as a safe haven to relieve distress and provide comfort, encouragement, and support; (c) using the attachment figure as a secure base to increase one’s sense of security; and (d) experiencing separation distress when the attachment figure is temporarily or permanently unavailable [48–53].

Many pet/owner relationships meet these four criteria and the majority of owners feel their pets are an integral part of their family [20, 30, 47, 54–56]. This exemplifies the expansion of the word “family”, moving beyond the traditional definition to a broader definition of family. In fact, many people view their pets as children [57–60] and think of themselves as ‘pet parents’. The definition of ‘parent’ is someone who brings up and cares for another; someone responsible for the physical, psychological, and financial support for those they care for [58, 61]. For many pet owners, therefore, ‘parent’ is an appropriate term to describe their pet caretaking role. Despite the importance of this relationship, it has often been overlooked when studying women dealing with breast cancer. Yet, because of the attachment many people diagnosed with breast cancer feel toward their pets, and the fact that many view their pets as family, we feel it imperative to explore this important relationship within the context of those experiencing breast cancer.

## Methods

An online, anonymous, cross-sectional survey was developed using Qualtrics (Qualtrics, Inc., Provo, UT, USA). The survey was designed, reviewed, and pilot tested by the co-investigators and their colleagues for ambiguity and/or potentially missing or inappropriate response options, with appropriate revisions made based on the pilot testing. The final survey (see Appendix) and study design were approved by the Colorado State University Institutional Review Board (IRB #3378). Survey participants were recruited between July 16, 2022 – November 1, 2022 via social media (Facebook, Twitter, Instagram) and breast cancer related events (e.g., Komen Race, etc.).

Adults (ages 18 and older), diagnosed with stages 0 (in situ) -IV breast cancer and currently the primary guardian of at least one dog or cat and owned the animal(s) for at least 6 months, were recruited for the study. Participant demographics were collected (type of pet owned (dog and/or cat); country, age, ethnicity, race, gender, education level, relationship status, number of children in the home, gross annual income, breast cancer stage, time since diagnosis, and if currently receiving treatment (yes/no and what type). If participants had more than one companion animal, they were asked to answer the animal related questions based on the companion animal to which they felt closest.

### Survey

The survey included several validated instruments including the Lexington Attachment to Pets Scale (LAPS), designed to measure attachment to one’s pet. The LAPS is a well validated attachment scale, with a reported internal consistency of 0.94 [62] that consists of 23 items and asks participants to rate each question on a 4-point Likert scale from 0 = strongly disagree to 4 = strongly agree. Examples of questions include “I believe my pet is my best friend” and “I love my pet because he/she never judges me”.

To assess participants’ physical, emotional, and functional wellbeing, they were given the FACT-G7, an instrument designed to assess symptoms and concerns related to a broad spectrum of cancers and has a reported reliability of α = 0.74 [63]. The FACT-G7 is a short version of the validated FACT-G, a 27-item measure of health-related quality of life that assesses physical, social/family, emotional, and functional well-being in the past 7 days. The FACT-G7 consists of 7 high-priority FACT-G items from the physical domain (“I have a lack of energy,” “I have nausea,” and “I have pain”), the emotional domain (“I worry that my condition will get worse”), and the functional domain (“I am able to enjoy life,” “I am sleeping well,” and “I am content with the quality of my life right now”). Items are rated on a 5-point Likert scale from 0 (not at all) to 4 (very much); with negative items reverse-scored. Total scores range between 0 and 28, with higher scores indicating better quality of life [63].

A modified version of the Medical Outcomes Study Social Support Survey (MOS-SS) was included to assess the perceived support participants receive from their companion animal. The MOS-SS is one of the most widely used instruments to assess social support [64]. The MOS-SS is comprised of 19 items scored on a Likert scale ranging from 1 (none of the time) to 5 (all of the time) and includes a global dimension of social support and four sub-dimensions: emotional/informational (8 items); positive social interaction (3 items); affection (3 items); and tangible (4 items) [64].

Our modified Medical Outcomes Study Social Support Survey Pet (MOS-SS-P) maintained the four sub-dimensions and included 20 items: emotional (7 items), positive social interaction (3 items), affection (3 items), and tangible (7 items). Examples of questions on the MOS-SS-P include “Ability to provide nonjudgmental support”, “Ability to listen to your most private worries and fears” and “Foster your desire to be physically healthy”. A total social support score was calculated by summing the responses of all 20 items. The scores for each of the four sub-dimensions were likewise created by summing the responses to the corresponding questions.

The Parenting Concerns Questionnaire (PCQ) was modified and used to reflect participants’ concerns related to caring for their pet, rather than their children, as a result of having cancer. The PCQ was developed for cancer patients to assess parental concerns related to their cancer. The original scale has 15 items with three factors, each containing five questions: practical impact of the disease on the child, emotional impact of the disease on the child, and concerns about the co-parent [65]. In the current study, we modeled our questions after the PCQ but changed the word ‘child’ to the word ‘pet’. Our PCQ-P instrument had 13 total questions (practical impact: 5 items, emotional impact: 5 items, concerns about co-parenting: 3 items).

Feelings of guilt related to caring for their pets, as a result of their cancer, were assessed by asking participants to indicate their agreement level to a series of seven statements using a 5-point Likert scale (1 = strongly disagree, 5 = strongly agree). Examples include “I often worry I will not be able to provide for my pet as I would like because of the cost of my cancer” and “I often worry I do not give my pet enough love and attention because of my cancer.”

Participants were also asked to report their perception of support from people around them (e.g., oncologist, veterinarian, mental health provider, partner, children, etc.) in terms of nurturing their relationship with their pet using a 5-point Likert scale (1 = very unsupportive to 5 = very supportive).

Several open-ended questions were included in the survey to allow for qualitative analysis. These included questions related to concerns/worries about caring for their pet, what resources they felt were lacking in caring for their pet, ways in which their pet hinders/hindered them, and biggest pet-related future worries. We additionally asked them to think about how their pet helped them during their hardest time of treatment, things they would like their medical team to know about their pet, and any advice they would give to pet owners diagnosed with breast cancer. Findings from the qualitative analysis will appear in a future manuscript.

### Analysis

Descriptive statistics were calculated to describe the participants. Because not all questions were answered by all participants, the total number of responses is noted when less than the total sample size. Multiple regression analyses were conducted to determine predictors of scores from the LAPS, MOS-SS-P, PCQ-P, overall pet-related guilt, and FACT-G7. Kruskal–Wallis tests were used to guide the selection of owner demographics as predictors for the FACT-G7 multiple regression model. Significance level (α) was set at *p* = 0.05 and all tests were two-tailed. Data were analyzed using SPSS (IBM, Armonk, NY, USA).

## Results

Responses from the online survey were assessed and those flagged as potential bots, duplicate responses, or participants who failed to complete at least 80% of the survey were eliminated, leaving a sample of 211 for analysis.

Our sample consisted of people diagnosed with breast cancer who identified as primarily White (181, 86%), non-Hispanic (152, 73%), female (207, 98%), and residing in the United States (188, 96%—Colorado specifically 74/188, 39%) (Table 1). Most participants were between 30 and 59 years of age (165, 78%), had at least a 4-year college degree (143, 69%), and a household income between $30,000–100,000 (122, 58%). The majority of participants reported being partnered/married (160, 76%). A total of 51 (25%) reported having 1 or more children 5 years of age or younger at home, 68 (33%) reported having 1 or more children 5–10 years of age, and 72 (35%) reported having 1 or more children 11–18 years of age (Table 1).

**Table 1:** Participant demographics

	N	%
Country (*n* = 195)		
Cats United States	188	96
Other	7	4
State in US (*n* = 188)		
Colorado	74	39
Other	114	61
Gender (211)		
Female	207	98
Non-binary / third gender	1	0.5
Other	1	0.5
Prefer not to say	2	1
Education level (*n* = 209)		
High school/GED or less	26	12
Vocational/trade school/2-year college	39	19
College (4-year)	81	39
Graduate School	62	30
Race (*n* = 211)		
African American/Black	6	3
Asian	5	2
Biracial/multiracial	1	0.5
Native American/Indigenous	10	5
Native Hawaiian/Pacific Islander	2	1
White/Caucasian	181	86
Prefer to not say	3	1
Prefer to self-describe	3	1
Age (*n* = 211)		
Under 30	27	13
30–39	61	29
40–49	61	29
50–59	43	20
60 and older	18	9
Prefer to not say	1	0.5
Income (*n* = 207)		
Less than $30,000	23	11
$30,000 to $49,999	41	20
$50,000 to $69,999	41	20
$70,000 to $99,999	40	19
$100,000 to $149,999	28	14
$150,000 or more	17	8
Prefer to not say	17	8
Children under 5 years of age (*n* = 206)		
0	154	75
1	39	19
2 or more	12	6
Prefer to not say	1	0.5
Children 5–11 years of age (*n* = 208)		
0	139	67
1	53	26
2 or more	15	7
Prefer to not say	1	0.5
Children 11–18 years of age (*n* = 208)		
0	135	65
1	46	22
2 or more	26	13
Prefer to not say	1	0.5
Cancer stage (*n* = 211)		
In situ (stage 0)	8	4
Stage I	53	25
Stage II	84	40
Stage III	44	21
Stage IV	14	7
Unknown	7	3
Other	1	0.5
When diagnosed (*n* = 211)		
Less than 2 months ago	12	6
More than 2 months but less than 12 months ago	81	38
1–2 years ago	66	31
2–4 years ago	29	14
4–9 years ago	23	11
Pets in the home		
Dogs (*n*=207)		
0	31	15
1	105	51
2	54	26
More than 2	17	8
Cats (*n*=207)		
0	104	50
1	57	28
2	36	17
More than 2	10	5

When queried about their current cancer stage, the largest number reported being in Stage II (84, 40%) and currently receiving treatment or care (190, 90%). The most common type of treatment or care they were currently receiving was chemotherapy (103/190, 54%), followed by medication (e.g., tamoxifen/Nolvadex, aromatase inhibitors) (95/190, 50%) and radiotherapy (74/190, 39%). A smaller number reported undergoing any type of surgical procedure (37/190, 19%) or receiving hospice or palliative care (13/190, 7%). Nearly one-half of participants reported being diagnosed less than 12 months ago (93, 44%) (Table 1).

Participants were asked how many dogs they currently have, to which the most common answer was one (105, 51%). When asked how many cats they currently have, half answered none, followed by 57 (28%) who reported one cat in the house. Attachment to the pet they felt closest to was measured with the Lexington Attachment to Pets Scale (LAPS). Scores ranged from 18 to 69 (X = 56.47, SD = 10.25, α = 0.92), with higher scores indicating greater pet attachment. Multiple linear regression was conducted on the total LAPS score to determine the impact of owner demographics (age, education, income, child status, relationship status) on pet attachment. The multiple regression model was significant (F(19) = 2.30, *p* = 0.003, *R*^2^ = 0.21). Significant predictors of LAPS scores included having children under the age of five in home, in which those with no children under five years of age (B = 11.03; *p* = 0.002), or one child under the age of five (B = 8.46; *p* < 0.030), reported higher attachment than those with two or more children under the age of five in the home. Additionally, those who reported having no children ages 11–18 in the home reported higher attachment than those with two or more children between the ages of 11–18 in the home (B = 5.34; *p* = 0.020). Having children between the ages of 5–11 had no significant impact on pet attachment.

Participants were asked to indicate how supportive people around them (e.g., partner, neighbors, oncologist, veterinarian, etc.) were in nurturing their relationship with their pet, using a 5-point Likert scale with 1 = very unsupportive and 5 = very supportive. The majority of participants reported feeling the people in their lives were somewhat or very supportive of their relationship with their pet: family (156/207, 75.4%), partner (147/197, 74.6%), mental health providers (140/201, 69.6%), friends (141/207, 68.1%), child(ren) (122/198, 61.6%), neighbors (111/204, 54.4%), veterinarian (109/201, 54.3%), and oncologist (106/205, 51.7%).

### Concerns about ‘pet parenting’

Concerns about ‘pet parenting’ were assessed using a modified form of the Parenting Concerns Questionnaire (PCQ) [65]. Our PCQ-P instrument had 13 total questions divided into three domains (Practical, Emotional, and Concerns about Co-parenting). The mean score for the total PCQ-P scale was 3.07 (SD = 1.13, α = 0.96). Responses to the individual items of the PCQ-P are presented in Table 2. We combined questions within each domain and the mean, standard deviations, and alpha coefficients for the domains were: Practical Impact: X = 3.08, SD = 1.10, α = 0.91, Emotional Impact: X = 3.00, SD = 1.19, α = 0.93, and Concerns about Co-parenting: X = 3.15, SD = 1.44, α = 0.94.

**Table 2:** Concerns about ‘pet parenting’ – scores from the Parenting Concerns Questionnaire (PCQ-P) items

	Not at all concerned		A little bit concerned		Somewhat concerned		Very concerned		Extremely concerned	
**Concerns about co-parent**										
There is no one to take good care of my pet if I die	53	25.2%	14	6.7%	26	12.4%	60	28.6%	57	27.1%
I do not have a responsible caregiver for my pet if I died	62	29.7%	19	9.1%	27	12.9%	54	25.8%	47	22.5%
There is no one who would be able to meet my pet’s emotional needs if I died	48	23.1%	19	9.1%	34	16.3%	65	31.3%	42	20.2%
**Parent practical impact**										
My illness is changing my pet’s routines	30	14.3%	35	16.7%	51	24.3%	54	25.7%	40	19.0%
I am not able to spend as much time with my pet as I would like	32	15.4%	35	16.8%	41	19.7%	61	29.3%	39	18.8%
My physical limits or low energy level are negatively affecting my pet	26	12.4%	36	17.1%	60	28.6%	61	29.0%	27	12.9%
Changes in my memory and attention are negatively affecting my pet	47	22.5%	34	16.3%	51	24.4%	54	25.8%	23	11.0%
My own mood, worries or emotions are negatively affecting my pet	32	15.2%	39	18.5%	56	26.5%	63	29.9%	21	10.0%
**Emotional impact**										
My pet seems worried about me	24	11.4%	34	16.2%	47	22.4%	65	31.0%	40	19.0%
My pet seems confused or upset by changes brought about because of my illness	36	17.2%	32	15.3%	55	26.3%	51	24.4%	35	16.7%
My pet gets upset when I talk about my illness	63	30.0%	29	13.8%	48	22.9%	37	17.6%	33	15.7%
My pet’s mental/emotional health is suffering because of my illness	53	25.4%	31	14.8%	43	20.6%	50	23.9%	32	15.3%
My pet is emotionally upset by my illness	43	20.5%	32	15.2%	50	23.8%	61	29.0%	24	11.4%

Multiple linear regression was conducted for each of the three domains (Practical, Emotional, and Concerns about Co-parenting) to determine the impact of owner demographics (age, education, income, child status, relationship status, when diagnosed, cancer stage, LAPS score). The multiple regression model for Practical Concerns of their cancer on their pet was significant (F(28)  = 2.26, *p* = 0.001, *R*^2^ = 0.30). Significant predictors of the Practical Impact domain score included having children between the ages of 5–11 in the home, in which those with no children between the ages of 5–11 reported lower concern than those with two or more children. Additionally, those who reported having no children ages 11–18 in the house reported lower concern than those with two or more children between the ages of 11–18 in the house. No other variables were significant predictors.

The multiple regression model for the Emotional Impact of their cancer on their pet was significant (F(28)  = 2.63, *p* < 0.001, *R*^2^ = 0.34). Significant predictors of the Emotional Impact domain score included having children between the ages of 5–11 in the home, in which those with no children between the ages of 5–11 reported lower concern than those with two or more children. Additionally, those who reported having no children ages 11–18 in the house reported lower concern than those with two or more children between the ages of 11–18 in the house. No other variables were significant predictors. The multiple regression model for Concerns about Co-parenting related to their pet had no significant predictors.

### Pet related support

Our adaptation of the MOS-SS (MOS-SS-P) was used to assess the support participants felt they receive from their companion animals. Our scale included 20 items (possible range = 20–100, α = 0.92) divided into four sub-dimensions: Emotional (possible range = 7–35, 7 items, α = 0.87); Social (possible range = 3–15, 3 items, α = 0.84); Affectionate (possible range = 3–15, 3 items, α = 0.84); and Tangible (possible range = 7–35, 7 items, α = 0.80). The total Social Support score and four sub-dimensions were calculated by summing the responses. The mean score for total Social Support was 79.90 (SD = 13.00, range 41–100). Mean scores for the sub-dimensions included: Emotional (M = 28.52, SD = 5.14, range = 14–35); Social (M = 12.32, SD = 2.49, range = 4–15); Affectionate (M = 12.92, SD = 2.38, range = 6–15); and Tangible (M = 26.14, SD = 5.32, range = 12–35) (Table 3).

**Table 3:** Perceived emotional, social, affectionate, and tangible support from pets

	None of the time		A little of the time		Some of the time		Most of the time		All of the time	
**Affectionate support**										
Make you feel loved	0	0.0%	7	3.3%	30	14.3%	50	23.8%	123	58.6%
Make you feel needed	1	0.5%	11	5.3%	32	15.3%	51	24.4%	114	54.5%
Offer you the opportunity to cuddle	1	0.5%	12	5.7%	25	11.9%	58	27.6%	114	54.3%
**Emotional support**										
Ability to share quiet time together	1	0.5%	8	3.8%	23	11.0%	66	31.6%	111	53.1%
Ability to listen to your most private worries and fears	4	1.9%	9	4.3%	41	19.5%	51	24.3%	105	50.0%
Provide a positive presence in the home	3	1.4%	7	3.3%	32	15.2%	64	30.5%	104	49.5%
Ability to provide nonjudgmental support	1	0.5%	17	8.1%	45	21.4%	61	29.0%	86	41.0%
Help you get your mind off things	1	0.5%	12	5.7%	38	18.2%	75	35.9%	83	39.7%
Ability to help you feel understood	4	1.9%	22	10.5%	51	24.3%	63	30.0%	70	33.3%
Ability to listen when you need to talk	2	1.0%	22	10.5%	38	18.1%	80	38.1%	68	32.4%
**Positive social interaction**										
A ‘partner’ to relax with	2	1.0%	10	4.8%	36	17.1%	69	32.9%	93	44.3%
A ‘partner’ to enjoy daily activities together with	0	0.0%	10	4.8%	38	18.1%	74	35.2%	88	41.9%
A ‘partner’ to play with	5	2.4%	17	8.1%	32	15.2%	69	32.9%	87	41.4%
**Tangible support**										
Foster your efforts to be active and move around	8	3.8%	9	4.3%	45	21.4%	58	27.6%	90	42.9%
Offer you the opportunity to care for another being	5	2.4%	23	11.0%	36	17.1%	60	28.6%	86	41.0%
Foster your desire to be physically healthy	7	3.3%	12	5.7%	45	21.5%	60	28.7%	85	40.7%
Foster your ability to maintain a regular schedule	3	1.4%	17	8.1%	37	17.6%	82	39.0%	71	33.8%
Foster your efforts to go outdoors	9	4.3%	17	8.1%	43	20.5%	76	36.2%	65	31.0%
Foster your social connections with other people	15	7.2%	31	14.8%	60	28.7%	69	33.0%	34	16.3%
Help you eat regularly	32	15.2%	37	17.6%	52	24.8%	55	26.2%	34	16.2%

Multiple linear regression was conducted for each of the four sub-dimensions (Emotional, Social, Affectionate, and Tangible support from their pets) to determine the impact of owner demographics (age, education, income, child status, relationship status, when diagnosed, cancer stage, LAPS score). The multiple linear regression predicting perceived Emotional support from their pets was significant (F(28)  = 5.05, *p* < 0.001, R^2^ = 0.49). The only significant predictor was LAPS score; those who reported higher attachment to their pet also reported more perceived Emotional support (B = 0.291; *p* < 0.001).

The multiple linear regression predicting the perceived Social support from their pets was significant (F(28)  = 3.75, *p* < 0.001, *R*^2^ = 0.42). The only significant predictor was LAPS score, whereby those who reported higher pet attachment also reported more Social support (B = 0.116; *p* < 0.001).

The same trend was seen in the multiple linear regression results pertaining to the Affectionate support from their pets (F(28) = 4.56, *p* < 0.001; *R*^2^ = 0.47) whereby the only significant predictor was LAPS score; those who reported higher pet attachment also reported more Affectionate support (B = 0.121; *p* < 0.001) and the multiple linear regression pertaining to the Tangible support from their pets (F(28) = 3.18, *p* < 0.001; *R*^2^ = 0.40). Higher LAPS scores predicted higher perceived Tangible support from their pets (B = 0.223; *p* < 0.001).

### Guilt

Feelings of guilt associated with caring for their pets while experiencing cancer were assessed by asking participants to rate their feelings to a series of seven pet-related guilt statements (Table 4). Possible range of scores was 7- 49. The range of scores in the sample was 8–35, α = 90. The mean of this scale was 3.49 (SD = 0.90).

**Table 4:** Agreement level with seven statements pertaining to guilt and their pets

	Strongly Disagree		Disagree		Neither Agree nor Disagree		Agree		Strongly Agree	
I feel guilty when I do not have the energy to fully engage with my pet because of my cancer	7	3.3%	28	13.3%	26	12.3%	95	45.0%	55	26.1%
I feel bad that I am unable to spend more time with my pet because of my cancer	11	5.3%	29	13.9%	34	16.3%	85	40.7%	50	23.9%
I feel bad when I have to put my own needs ahead of my pet because of my cancer	6	2.9%	36	17.1%	33	15.7%	88	41.9%	47	22.4%
I often worry I will not be able to provide for my pet as I would like because of the cost of my cancer	17	8.1%	40	19.0%	40	19.0%	71	33.6%	43	20.4%
I often worry I do not give my pet enough love and attention because of my cancer	15	7.1%	41	19.4%	27	12.8%	86	40.8%	42	19.9%
I often worry I will not be able to provide for my pet as I would like because of the cost of my cancer	12	5.7%	60	28.7%	34	16.3%	71	34.0%	32	15.3%
I often worry I am not as good a pet guardian as I should be because of my cancer	8	3.8%	48	22.7%	50	23.7%	83	39.3%	22	10.4%

Multiple linear regression was conducted on the total guilt score to determine the impact of owner demographics (age, education, income, child status, relationship status, when diagnosed, cancer stage, LAPS score) on feelings of guilt. The multiple linear regression was significant (F(28) = 2.06, *p* = 0.003, *R*^2^ = 0.28). The significant predictors included LAPS score, whereby those who reported higher attachment also reported more guilt (B = 0.030; *p* < 0.001), and time of diagnosis. Those diagnosed less than 2 months ago (B = 1.134; *p* = 0.003) or 2–12 months ago (B = 0.831; *p* = 0.003) were more likely to report higher levels of guilt than those diagnosed four years ago or more.

### Quality of life

Quality of Life was assessed using the FACT-G7 [63]. Scores ranged from 2 to 28 (M = 13.73, SD = 4.99) with a higher score indicating greater quality of life. Cronbach’s alpha was 0.70, comparable to that reported in previous studies [66].

Exploratory Kruskal–Wallis analyses were used to determine which owner demographics (age, education, income, child status, relationship status, when diagnosed, cancer stage) to include in the multiple linear regression model to predict Quality of Life (Table 5). No demographics, with the exception of when owners were diagnosed, were significantly associated with FACT-G7 scores (Table 3). Therefore, this was the only demographic variable included in the model. A multiple linear regression was conducted to determine predictors of Quality of Life, using FACT-G7 scores as the dependent variable. Predictor variables in the model included the Guilt Scale score, the total MOS-SS-P score, the total PCQ-P score, and when owners were diagnosed with cancer.

**Table 5:** Kruskal–Wallis test results assessing the association between FACT-G7 and owner demographics (*n* = 211)

	**H (df)**	***P***
Age	3.67 (4)	= 0.452
Education	6.93 (4)	= 0.140
Income	9.82 (4)	= 0.081
Relationship status	3.13 (4)	= 0.077
When diagnosed	11.44 (4)	= 0.022
Cancer stage	4.21 (4)	= 0.379
Children under 5	2.72 (3)	= 0.437
Children 5–11	0.86 (3)	= 0.834
Children 12–18	6.53 (3)	= 0.089

The multiple linear regression was significant (F(7) = 13.91, *p* < 0.001, *R*^2^ = 0.33). The significant predictors included Guilt scores (B = -0.296; *p* < 0.001), MOS-SS-P scores (B = 0.064; *p* = 0.007), and PCQ-P scores (B = -1.000; *p* = 0.002). Time since diagnosis was not a significant predictor. Higher guilt, higher parental concerns, and lower perceived support from their pet all predicted lower quality of life (Table 6).

**Table 6:** Results of the multiple linear regression model predicting Quality of Life (FACT-G7) as a function of MOS-SS scores, guilt scores, PCQ-pet scores, and time since diagnosis

ANOVA								
Model	Sum of Squares	df	Mean Squares	F	Sig			
Regression Residual Total	1694.38 3514.75 5209.12	7 202 209	247.95 9.13	13.91	< 0.001			
Coefficients* (Dependent Variable: FACT-G7)								
							95% confidence interval	
Variable			Coefficient (B)	Std. Error	t	Sig	Lower bound	Upper bound
(Constant)			19.19	2.12	9.06	< .001	15.01	23.37
Guilt scale score			-0.30	.06	-4.96	< .001	-.41	-.18
MOS-SS full scale score			0.06	.02	2.70	.007	.02	.11
PCQ full scale score			-1.00	.32	-3.13	.002	-1.63	-.37

## Discussion

While breast cancer is still the most common cancer among female-identifying persons, advances in treatment have led to improved patient survival rates [67]. With the 5-year survival rate in high income countries now reaching 85–90% [68], there has been an increased focus on psychosocial factors associated with survivorship [69–72]. Psychosocial factors, including depression, anxiety, and environmental challenges, can negatively affect the effectiveness of the health care, quality of life, and mental well-being among people diagnosed with breast cancer [73, 74]. According to the World Health Organization (WHO), mental health is an integral component of health and well-being [75]. Despite the large number of studies that have explored different types of supportive care to enhance the well-being of people diagnosed with breast cancer [76], none have focused on the relationship and care of their companion animals.

Pet ownership, however, is at an all-time high; about 70% of U.S. households include at least one pet [19] with Millennials (people currently between 23–41 years of age) owning more pets than any other age group in the U.S. [77]. The bond between humans and companion animals has been defined as a mutually beneficial and dynamic relationship influenced by behaviors essential to the health and well-being of both [78]. Attachment theory has been used to explain this bond with several studies suggesting that higher attachment to pets predicts a number of positive outcomes [79, 80].

Numerous studies suggest pets have a positive impact on owners’ mood and well-being [23, 27, 31, 81–86], provide social support and companionship, and can reduce loneliness [30, 87–89]; all of which can protect against adverse effects of stressors [90]. Interacting with dogs has been shown to improve owners’ physical health [91], reduce doctor visits [87], increase survival after cardiovascular events [92, 93] and reduce cardiovascular disease risk [94, 95]. Results of these studies led the American Heart Association to suggest that pet ownership (and dogs in particular) may reduce the risk for cardiovascular disease [95]. Additional studies report that cardiovascular activity (such as walking a dog) may reduce the risk of breast cancer recurrence [96, 97].

Pets have also been linked to a reduction in cortisol and an increase in oxytocin concentrations [98–102]. Dogs in particular have been linked with increased exercise and positive social interactions [103–110].

In our study, we found that the majority of participants reported feeling very attached to their pets. Those without children reported higher pet attachment than those with children, similar to results reported by Volsche [111]. Most participants in our study also reported feeling they receive a great deal of support from their pets, regardless of the their age, education, income, child or relationship status, or when they were diagnosed. In fact, the only factor that predicted participants’ perceived support from their pet was attachment level; people who felt more attached to their pet reported receiving more support. When we explored different types of support they received from their pet, we found that affectionate support was rated the highest. Most participants (approximately 80%) reported feeling loved or needed by their pets most or all the time. When asked about emotional support, the areas rated as the highest included ‘ability to share quiet time together’ (most or all the time: 85%), ‘provide a positive presence in the home’ (81%), and ‘ability to listen to their most private worries and fears’ (74%). For positive social interaction support, the highest rated items included ‘a partner to relax with’ (most or all the time: 77%) and ‘a partner to enjoy daily activities together with’ (77%). Tangible support was endorsed less often, but still felt important by a number of participants. The highest rated types of tangible support included ‘fostering efforts to be active and move around’ (most or all the time: 71%), ‘an opportunity to care for another being’ (70%), and ‘a desire to be physically healthy’ (70%). Given the research on the positive effects of social support on breast cancer patients and survivors [76, 112, 113], it is important to note that the majority of participants in our study felt they obtain support from their pets in a multitude of ways.

Despite the high levels of support participants reported receiving from their pets, only about half of our sample felt their veterinarians or oncologists were supportive of their relationship with their pet. This might be due to fears pertaining to zoonotic risks. The main concern many professionals have about cancer patients owning pets is the risk of zoonotic infections, especially for people who are immunocompromised receiving immunosuppressive treatment [114]. Zoonoses are infections that are transmitted between vertebrate animals and humans [115]. Despite widespread fears related to zoonoses, the risk of people receiving cancer treatment acquiring a zoonotic infection is not known, partially due to the fact that cases in which immunocompromised persons are affected by zoonotic infections tend to be sporadic and non-reportable infections [116]. The incidence, however, is felt to be low [114] and for many people diagnosed with cancer, the benefits of pets may outweigh the risks [116–118]. There are several studies that suggest that immunocompromised persons are not at any additional risk from pet contact than the general population [119–123]. Yet many clinicians treating immunocompromised patients feel uncomfortable discussing zoonotic risks, and as a result, most are unaware of pet ownership among their patients [124–127]. To facilitate safety, the issue of pet ownership and ways to reduce zoonotic risks should be addressed by a multidisciplinary team that includes veterinarians so that animal factors such as species and husbandry-related aspects of pet contact can be appropriately communicated [114, 128].

Regardless of whether the topic of pets is discussed by medical professionals, it is clear that many people experiencing cancer have pets, and not including them in conversations and psychosocial support plans can leave those unaware of zoonotic risks and struggling with pet-related concerns and guilt. When assessing ‘parental’ concerns related to pets, we found the only significant predictor of participants’ emotional, practical, and co-parenting concerns was whether they had children. Participants with children expressed more concern about each of these areas than those without children. It is possible that participants with children feel more concern about the impact of their cancer on their pets because they already have at least one other dependent relying on them for care. Caring for dependents, whether they are children or pets, can take the form of direct (e.g., bathing/grooming, feeding, medical care) or indirect (e.g., play, and other activities that facilitate emotional and cognitive development) [59]. Both types of care can be a struggle for people going through breast cancer treatment [129]. Many cancer treatments have significant side effects that impact the ability to care for children or accomplish household tasks [130] which can negatively impact wellbeing, cause feelings of guilt, depression and anxiety [14, 65, 131–137]. Given these stressors, parents may feel additional strain and concern when it comes to trying to meet their pet’s needs.

When we asked people diagnosed with breast cancer about their concerns regarding ‘parenting’ their pets, the area of most concern pertained to finding a caretaker for their pet if something were to happen to them. Examples included ‘there is no one to take good care of my pet if I die’ (rated as very or extremely concerned: 56%), ‘there is no one who would be able to meet my pet’s emotional needs if I died’ (very or extremely concerned: 51%), and ‘I do not have a responsible caregiver for my pet if I died’ (very or extremely concerned: 49%). Several practical impacts were also reported as concerning by a large number of participants. Examples included ‘my illness is changing my pet’s routines’ (rated as very or extremely concerned: 45%), and ‘I am not able to spend as much time with my pet as I would like’ (very or extremely concerned: 48%).

In Inhestern et al.’s [13] study of cancer survivors with minor children, 31% of survivors reported being somewhat to extremely concerned about the emotional impact of cancer on their children, compared to 57% of our sample who reported being somewhat to extremely concerned about the emotional impact of their cancer on their pets. In terms of practical impact, Inhestern et al. found that 28% of survivors were somewhat to extremely concerned, compared to 61% of our sample. When assessing concerns related to co-parenting, Inhestern reported 18% of survivors were somewhat to extremely concerned, while our study found that 62% of participants reported feeling somewhat to extremely concerned about the emotional impact of cancer on their pet [13]. Concern means reported by Muriel (2012) for the three subscales were emotional: 2.38 (compared to our study’s mean of 3.00); practical: 2.64 (compared to our study’s mean of 3.08), and co-parenting: 1.95 (compared to our study’s mean of 3.15). Although some of the questions in our study were worded slightly different than the original PCQ, these results suggest that people experiencing cancer are concerned about their pets in ways that mirror or exceed concerns about children.

Concern can translate into guilt, and many participants reported feeling guilty about their ability to care for their pets. We found that over 50% of participants reported agreeing or strongly agreeing to 5 out of 7 potential areas of guilt. Guilt refers to an unpleasant emotional state associated with one’s behaviors, thoughts or intentions and is based on the possibility that one may have wronged others, regardless of whether any actual physical, psychological, or emotional harm has taken place [138–140]. A recent study by Kogan et al. [141] found that people with pets report guilt scores similar to those reported in studies assessing parents and their human children, with female pet owners reporting higher levels of guilt than male pet owners. Pet-related guilt is often unrecognized, yet, similar to parental guilt [142–144] it may lead to feelings of anxiety depression and poor psychological health [141]. The guilt items endorsed the most frequently in the current study included ‘I feel guilty when I do not have the energy to fully engage with my pet because of my cancer’ (agree or strongly agree: 71%), ‘I feel bad that I am unable to spend more time with my pet because of my cancer’ (agree or strongly agree: 65%), and ‘I feel bad when I have to put my own needs ahead of my pet because of my cancer’ (64%).

We found that people diagnosed with breast cancer experienced heightened levels of guilt, along with higher ‘pet-parent' concerns and lower perceived support from their pet, predicted lower quality of life. Care planning and conversations that include the topic of pets might help people diagnosed with breast cancer with their pet-related concerns and guilt. Our findings highlight the increased distress people feel in trying to meet their pets’ needs during the initial months following cancer diagnosis. As such, the assessment of pet ownership and the strength of their pet care support system early in the treatment trajectory may create opportunities to validate the concerns of people diagnosed with breast cancer and open dialogues about ways to enhance their pet’s psychosocial support care team. When available, resources such as Cancer*Care’s Pet Assistance and Wellness* Program (https://www.cancercare.org/paw) can help alleviate financial and emotional distress by offsetting the expenses of daily pet care or veterinary services, and providing educational materials to foster communication with medical and veterinary providers about their pet parenting needs. Additionally, online interactive pet services resource guides (https://viewer.mapme.com/pets-and-cancer) can help those going through cancer to easily locate providers to help with pet socialization and exercise, grooming, boarding, transportation, and free or low-cost veterinary providers.

Just as parents diagnosed with cancer often struggle with feelings of distress and guilt [13, 131], it appears that many who own pets share similar feelings. Breast cancer often leaves people less able to care for their family which can cause feelings of guilt and a tendency to put others’ needs ahead of their own [131]. Many people diagnosed with breast cancer struggle with depression and anxiety [134, 135, 137], and having dependents can exacerbate these negative effects [14, 65]. Unfortunately, despite the need for family-centered psychological support, it remains rare and underutilized [135, 145–147].

We suggest that family-centered psychological support not only be made more accessible, but that it includes discussion and plans pertaining to companion animals. Not only should people diagnosed with cancer be asked whether they have children and informed about appropriate support services [145, 148, 149], we propose they should be asked about their pets. Many common tasks associated with caring for a pet, such as dog walking, litter box cleaning, carrying pet food, or even playing, may be challenging during cancer treatment and recovery. Helping people think through alternative options for pet care and acknowledging their feelings of guilt related to not being able to care for their pet the way they are accustomed to may help mitigate negative repercussions.

In conclusion, studies have suggested that caregiving roles can impact overall health and well-being [150] and many people diagnosed with cancer feel torn between their roles as patient and care provider [17, 65, 131, 151]. Our study suggests that for many experiencing breast cancer, these challenges extend to their companion animals, negatively impacting their well-being. It is important that medical and veterinary professionals, as well as breast cancer supporters and caregivers, acknowledge and validate the bond between people and their pets by including companion animals in breast cancer psychosocial support and care planning. These discussions could be aided through the development of research-driven intervention strategies and online, freely accessible targeted tools.

Limitations of this study are those inherent in online surveys, including the potential bias of those who chose to participate. It is possible that participants in this study are not representative of people diagnosed with breast cancer. Furthermore, because this is a new line of research, and there are no established scales to measure pet related support or ‘parenting’ concerns, we had to modify the MOSS-SS and the PCQ. Yet, we found both of these modified scales demonstrated adequate reliability (α = 0.87 and α = 0.96 respectively). Future studies to replicate these findings and expand this line of research can further validate these two new scales as well as enlarge the population to include non-female-identifying persons and people coping with different types of cancers. These studies can help lay the foundation needed to develop intervention strategies that can be widely implemented to ensure all medical professionals have easily accessible tools to help support the relationship people experiencing cancer share with their companion animals.

### Supplementary Information

**Additional file 1.** A One health approach to supporting breast cancer survivors and their companion animals survey.

## Data Availability

The datasets used and/or analyzed during the current study are available from the corresponding author on reasonable request.
