# Supplementary material for: Breast cancer treatment and recovery: pets’ roles as emotional buffers and stressors
Source: BMC Womens Health. 2023 Oct 17;23:540. doi: 10.1186/s12905-023-02662-z (PMC10583337; doi:10.1186/s12905-023-02662-z)
Supplement: Supplementary file 1 — Additional file 1. A One health approach to supporting breast cancer survivors and their companion animals survey. [file 12905_2023_2662_MOESM1_ESM.docx]

A One Health Approach to Supporting Breast Cancer Survivors and Their Companion Animals Survey

**A One Health Approach to Supporting Breast Cancer Survivors and their Companion Animals**


We are researchers from Colorado State University and the University of Colorado Cancer Center. We are conducting a study to explore the benefits and challenges breast cancer survivors encounter in providing for their companion animals’ present and future care needs.

We are looking for adult women who have been diagnosed with stages 0-IV breast cancer and are the current primary guardian of at least one dog or cat, and have owned the animal(s) for at least 6 months.

If you fit these criteria, we invite you to complete the following short (10-15 minute) anonymous survey. All participants will have the opportunity at the end of the survey to enter a raffle for a $100 Chewy gift card.

Your participation in this research is voluntary. If you decide to participate in the study, you may stop the survey and close the website at any time, without penalty. We will not collect your name or personal identifiers. When we report and share the data to others, we will combine the data from all participants. While there are no direct benefits to you, the knowledge we gain on survivors’ needs related to their companion animals will allow better support for future breast cancer survivors.

It is not possible to identify all potential risks in research procedures, but the researcher(s) have taken reasonable safeguards to minimize any known and potential (but unknown) risks. If you have any questions about the research, please contact Lori Kogan at lori.kogan@colostate.edu. If you have any questions about your rights as a volunteer in this research, contact the CSU IRB at: RICRO_IRB@mail.colostate.edu; 970-491-1553. If you consent to complete this survey, please click “Yes I consent” below, to begin the survey.

- Yes, I consent to participating in this survey
- No, I do not consent to participating in this survey

**Please tell us a little bit about you and your companion animals.**

How many dogs do you currently have?

▼ 0 ... 10

How many cats do you currently have?

▼ 0 ... 10

What best describes you? I was diagnosed:

- 2-12 months ago
- 1-2 years ago
- 2-4 years ago
- 4-9 years ago
- 10 or more years ago

End of Block: Please tell us a little bit about you and your companion animals.

Start of Block: Block 1

**For the rest of this survey, please answer all pet-related questions about the dog or cat you feel the closest to.**

Please indicate your agreement with the following statements about your pet.

|  | Strongly Disagree | Somewhat Disagree | Somewhat Agree | Strongly Agree |
| --- | --- | --- | --- | --- |
| My pet means more to me than any of my friends. |  |  |  |  |
| Quite often I confide in my pet. |  |  |  |  |
| I believe that pets should have the same rights and privileges as family members. |  |  |  |  |
| I believe my pet is my best friend. |  |  |  |  |
| Quite often, my feelings toward people are affected by the way they react to my pet. |  |  |  |  |
| I love my pet because he/she is more loyal to me than most of the people in my life. |  |  |  |  |
| I enjoy showing other people pictures of my pet. |  |  |  |  |
| I think my pet is just a pet. |  |  |  |  |
| I love my pet because he/she never judges me. |  |  |  |  |
| My pet knows when I’m feeling bad. |  |  |  |  |
| I often talk to other people about my pet. |  |  |  |  |
| My pet understands me. |  |  |  |  |
| I believe that loving my pet helps me stay healthy. |  |  |  |  |
| Pets deserve as much respect as humans do. |  |  |  |  |
| My pet and I have a very close relationship. |  |  |  |  |
| I would do anything to take care of my pet. |  |  |  |  |
| I play with my pet often. |  |  |  |  |
| I consider my pet to be a great companion. |  |  |  |  |
| My pet makes me feel happy. |  |  |  |  |
| I feel that my pet is part of my family. |  |  |  |  |
| I am not very attached to my pet. |  |  |  |  |
| Owning a pet adds to my happiness. |  |  |  |  |
| I consider my pet to be a friend. |  |  |  |  |

Please indicate your agreement level to the following statements.

|  | Strongly Disagree | Disagree | Somewhat Disagree | Neither Agree nor Disagree | Agree | Strongly Agree |
| --- | --- | --- | --- | --- | --- | --- |
| I often worry I am not as good a pet guardian as I should be because of my cancer. |  |  |  |  |  |  |
| I often worry I will not be able to provide for my pet as I would like because of the cost of my cancer. |  |  |  |  |  |  |
| I feel bad when I have to put my own needs ahead of my pet because of my cancer. |  |  |  |  |  |  |
| I feel bad that I am unable to spend more time with my pet because of my cancer. |  |  |  |  |  |  |
| I often worry I do not give my pet enough love and attention because of my cancer. |  |  |  |  |  |  |
| I feel guilty when I do not have the energy to fully engage with my pet because of my cancer. |  |  |  |  |  |  |
| I often worry I will not be able to provide for my pet as I would like because of the cost of my cancer. |  |  |  |  |  |  |

Below is a list of statements that other people with your illness have said are important. Please indicate your response as it applies to the past 7 days.

|  | Not at all | A little bit | Somewhat | Quite a bit | Very much |
| --- | --- | --- | --- | --- | --- |
| I have a lack of energy. |  |  |  |  |  |
| I have pain. |  |  |  |  |  |
| I have nausea. |  |  |  |  |  |
| I worry that my condition will get worse. |  |  |  |  |  |
| I am sleeping well. |  |  |  |  |  |
| I am able to enjoy life. |  |  |  |  |  |
| I am content with the quality of my life right now. |  |  |  |  |  |

End of Block: Block 1

Start of Block: Please answer the following questions about your pet.

Please tell us about your concerns or worries related to the care of your pet's daily needs while you are dealing with cancer.

________________________________________________________________

What resources do you feel are lacking in helping you care for your pet during your treatment or recovery?

________________________________________________________________

In thinking back on the hardest moments of your treatment, how did your pet hinder you?

________________________________________________________________

In thinking about your future health, what are your biggest worries for your pet?

________________________________________________________________

People sometimes look to their pets for companionship, assistance, and other types of support. Thinking of your pet(s), how often does your pet(s) provide the following types of support? If you have more than one pet, please answer the questions about the pet you feel closest to.

|  | None of the time | A little of the time | Some of the time | Most of the time | All of the time |
| --- | --- | --- | --- | --- | --- |
| Ability to listen when you need to talk |  |  |  |  |  |
| Provide a positive presence in the home |  |  |  |  |  |
| Ability to help you feel understood |  |  |  |  |  |
| Ability to provide nonjudgmental support |  |  |  |  |  |
| Ability to listen to your most private worries and fears |  |  |  |  |  |
| Ability to share quiet time together |  |  |  |  |  |
| Foster your efforts to be active and move around |  |  |  |  |  |
| Help you eat regularly |  |  |  |  |  |
| Foster your desire to be physically healthy |  |  |  |  |  |
| Foster your ability to maintain a regular schedule |  |  |  |  |  |

People sometimes look to their pets for companionship, assistance, and other types of support. Thinking of your pet(s), how often does your pet(s) provide the following types of support? If you have more than one pet, please answer the questions about the pet you feel closest to.

|  | None of the time | A little of the time | Some of the time | Most of the time | All of the time |
| --- | --- | --- | --- | --- | --- |
| Foster your efforts to go outdoors |  |  |  |  |  |
| Foster your social connections with other people |  |  |  |  |  |
| Offer you the opportunity to care for another being |  |  |  |  |  |
| Make you feel needed |  |  |  |  |  |
| Make you feel loved |  |  |  |  |  |
| Offer you the opportunity to cuddle |  |  |  |  |  |
| A ‘partner’ to enjoy daily activities together with |  |  |  |  |  |
| A ‘partner’ to relax with |  |  |  |  |  |
| A ‘partner’ to play with |  |  |  |  |  |
| Help you get your mind off things |  |  |  |  |  |

In thinking back on the hardest moments of your treatment, how did your pet help you?

________________________________________________________________

If there was something you would like your medical team to know about your pet, what might that be?

________________________________________________________________

Please share advice that you would give to other women diagnosed with breast cancer who have a pet:

________________________________________________________________

End of Block: Please answer the following questions about your pet.

Start of Block: Please answer the following questions about your pet.

| Page Break |  |
| --- | --- |

The following questions ask about some areas that may concern you about the pet you feel closest to. Please rate how concerned you have been about each of these things since your diagnosis.

|  | Not at all concerned | A little bit concerned | Somewhat concerned | Very concerned | Extremely concerned |
| --- | --- | --- | --- | --- | --- |
| My own mood, worries or emotions are negatively affecting my pet. |  |  |  |  |  |
| My pet gets upset when I talk about my illness. |  |  |  |  |  |
| Changes in my memory and attention are negatively affecting my pet. |  |  |  |  |  |
| My pet’s mental/emotional health is suffering because of my illness. |  |  |  |  |  |
| My illness is changing my pet’s routines. |  |  |  |  |  |
| There is no one to take good care of my pet if I die. |  |  |  |  |  |
| My pet is emotionally upset by my illness. |  |  |  |  |  |
| My physical limits or low energy level are negatively affecting my pet. |  |  |  |  |  |
| My pet seems worried about me. |  |  |  |  |  |

| Page Break |  |
| --- | --- |

The following questions ask about some areas that may concern you about the pet you feel closest to. Please rate how concerned you have been about each of these things since your diagnosis.

|  | Not at all concerned | A little bit concerned | Somewhat concerned | Very concerned | Extremely concerned |
| --- | --- | --- | --- | --- | --- |
| I am not able to spend as much time with my pet as I would like |  |  |  |  |  |
| My pet seems confused or upset by changes brought about because of my illness |  |  |  |  |  |
| My pet is not providing me with enough emotional support |  |  |  |  |  |
| I do not have a responsible caregiver for my pet if I died |  |  |  |  |  |
| There is no one who would be able to meet my pet’s emotional needs if I died |  |  |  |  |  |

Please indicate how supportive the following people were/are in nurturing your relationship with your companion animal during your cancer experience:

|  | Very Unsupportive | Somewhat Unsupportive | Neutral | Somewhat Supportive | Very Supportive |
| --- | --- | --- | --- | --- | --- |
| Partner/spouse |  |  |  |  |  |
| Children |  |  |  |  |  |
| Family |  |  |  |  |  |
| Friends |  |  |  |  |  |
| Neighbors |  |  |  |  |  |
| Doggy day care |  |  |  |  |  |
| Dog walker |  |  |  |  |  |
| Pet sitter |  |  |  |  |  |
| Oncologist |  |  |  |  |  |
| Other medical professionals (excluding oncologist) |  |  |  |  |  |
| Veterinarian |  |  |  |  |  |
| Mental health professionals |  |  |  |  |  |

Please indicate any other groups or individuals who have supported your relationship with your pet during your cancer experience: (please describe)

________________________________________________________________

What specific ways have your support networks (family, friends, vet, etc.) helped you care for your pet?

________________________________________________________________

End of Block: Please answer the following questions about your pet.

Start of Block: Please answer the following questions about yourself.

**Please answer the following questions about yourself.**

Please indicate what country you live in:

- United States
- Other (please specify): __________________________________________________

What state do you live in?

▼ Alabama ... I don't live in the United States

Please indicate your age:

▼ Under 30 years of age ... Prefer to not say

How do you identify yourself?

▼ Male ... Prefer to not say

How do you identify yourself?

- African American/Black
- Asian
- Biracial/multiracial
- Middle Eastern
- Native American/Indigenous
- Native Hawaiian/Pacific Islander
- White/Caucasian
- I prefer to not say
- I prefer to self-describe: __________________________________________________

What is your ethnicity?

- Hispanic/Latinx
- Not Hispanic/Latinx
- I prefer to not say

What is your education level?

▼ Less than high school ... I prefer to not say

Please indicate your gross annual income:

▼ Less than $10,000 ... I prefer to not say

Please indicate the number of children under 5 years of age in your home:

▼ 0 ... Prefer to not say

Please indicate the number of children between 5-10 years of age in your home:

▼ 0 ... Prefer to not say

 Please indicate the number of children between 11-18 years of age in your home:

▼ 0 ... Prefer to not say

Please indicate your marital/relationship status:

- Single
- Partnered/married
- Divorced
- Widowed
- Other (specify): __________________________________________________
- I prefer to not say

Please indicate your cancer stage:

- in-situ (stage 0)
- Stage I
- Stage II
- Stage III
- Stage IV
- Unknown
- Other

Are you currently receiving treatment or care?

- Yes
- No

What type of treatment or care are you receiving? (select all that apply)

- Chemotherapy (e.g., 5-fluoruracil, paclitaxel, doxorubicin, cisplatin)
- Radiotherapy
- Prescription pills (e.g., tamoxifen/Nolvadex, aromatase inhibitors)
- Hospice or palliative care
- Any surgical procedures

Thank you for your time. When you click on the arrow at the bottom right, your responses will be saved and you will automatically be directed to a new webpage where you can enter your contact information for the $100 Chewy gift card. 

End of Block: Please answer the following questions about yourself.
